# Supplementary material for: Exploring the BiFeO3-PbTiO3-SrTiO3 Ternary System to Obtain Good Piezoelectrical Properties at Low and High Temperatures
Source: Materials (Basel). 2023 Oct 24;16(21):6840. doi: 10.3390/ma16216840 (PMC10649841; doi:10.3390/ma16216840)
Supplement: Supplementary file 1 [file materials-16-06840-s001.zip › materials-2607319-supplementary.pdf]

Supplementary Information

Table S1. Relative density and piezoelectric propertie.

	**Composition**	**Type**	**Relative Density (%)**	**d_{33} (pC/N)**
I	0.40BFO-0.20PT-0.40ST	C	98	0
	0.45BFO-0.20PT-0.35ST	PC	98	25
	0.50BFO-0.20PT-0.30ST	PC	97	140
	0.55BFO-0.20PT-0.25ST	PC	97	105
	0.60BFO-0.20PT-0.20ST	PC	-	81
	0.70BFO-0.20PT-0.10ST	Rh	-	28
	0.80BFO-0.20PT-0.00ST *	Rh	95	30
II	0.70BFO-0.00PT-0.30ST *	PC	-	60
	0.55BFO-0.15PT-0.30ST	PC	-	75
	0.50BFO-0.20PT-0.30ST	PC	97	140
	0.45BFO-0.25PT-0.30ST	PC	-	250
	0.40BFO-0.30PT-0.30ST	PC	98	145
III	0.40BFO-0.25PT-0.35ST	C	-	0
	0.50BFO-0.15PT-0.35ST	PC	97	120
	0.55BFO-0.10PT-0.35ST	C	97	0
	0.60BFO-0.15PT-0.25ST	PC	-	77
	0.50BFO-0.25PT-0.25ST	PC	-	80
	0.70BFO-0.10PT-0.20ST	Rh	95	60
	0.70BFO-0.20PT-0.10ST	Rh	96	28

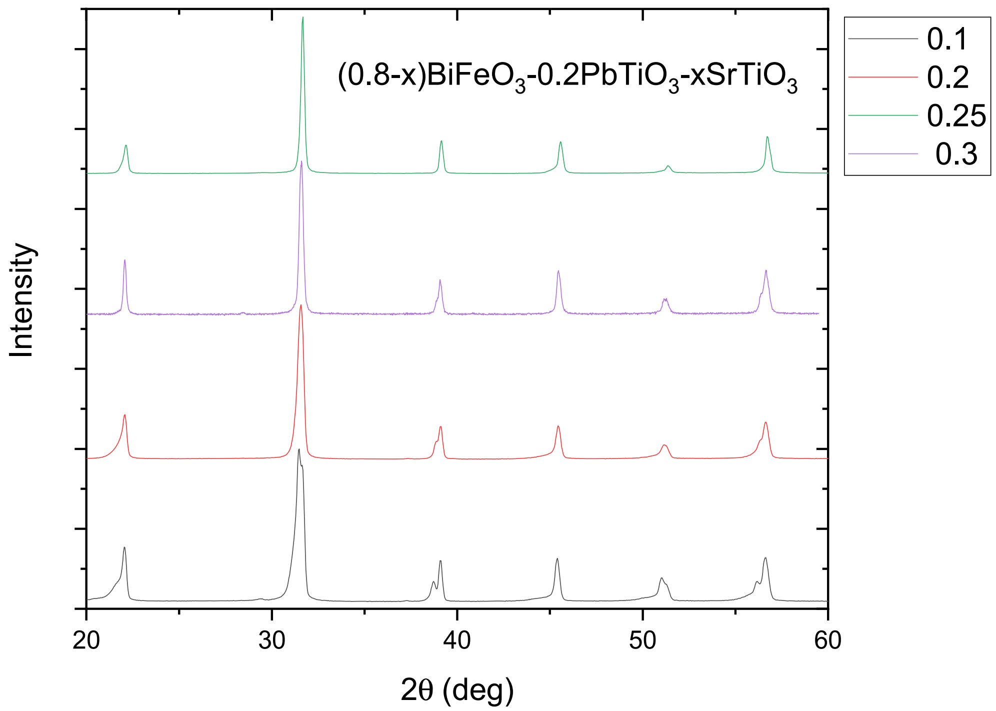

Figure S1. XRD patterns.
